# Spectral EEG-guided adaptive neuromodulation for social anxiety disorder, performance-only subtype: a case report

**DOI:** 10.3389/fpsyt.2026.1816923

**Published:** 2026-06-04

**Authors:** Mark Odron, Yatharth Mahajan, Vipul Reddy, Krrishika Saxena, Charles Vigilia, Brianna Dela Cruz, Jayleen Lu, Nisha Thunga, Kenneth Blum, David Baron, Keerthy Sunder

**Affiliations:** 1 Division of Clinical Neuromodulation Research, Karma TMS, Palm Springs, CA, United States; 2 University of California, Berkeley, Berkeley, CA, United States; 3 Sunder Foundation, Palm Springs, CA, United States; 4 Division of Addiction Research and Education, Center for Sports and Mental Health, Western University of Health Sciences, Pomona, CA, United States; 5 Department of Psychiatry, University of Vermont, Burlington, VT, United States; 6 Institute of Psychology, Eotvos Loránd University, Budapest, Hungary; 7 Department of Psychiatry, Wright University Boonshoft School of Medicine, Dayton, OH, United States; 8 Centre for Genomics and Applied Gene Technology, Institute of Integrative Omics and Applied Biotechnology, Nonakuri, West Bengal, India; 9 Department of Psychiatry, Stanford University School of Medicine, Palo Alto, CA, United States; 10 Department of Psychiatry, Riverside School of Medicine, University of California, Riverside, Riverside, CA, United States

**Keywords:** dorsolateral prefrontal cortex (DLPFC), Liebowitz social anxiety scale (LSAS), neuromodulation, personalized repetitive transcranial magnetic stimulation (PrTMS), social anxiety disorder (SAD), social phobia inventory (SPIN), spectral electroencephalography (EEG)

## Abstract

Social anxiety disorder (SAD) is often complicated by anticipatory anxiety, hyperarousal, and variability in response to first-line treatments such as cognitive behavioral therapy (CBT) and pharmacotherapy. These limitations highlight the need for adjunctive modalities capable of enhancing the magnitude and durability of treatment response. This case report explores outcomes following spectral EEG-guided personalized repetitive transcranial magnetic stimulation (PrTMS) in a patient with SAD, performance-only subtype. Weekly psychometric assessments revealed reductions in social anxiety intensity, avoidance behaviors, and negative self-appraisal, along with improvements in mood and daily function. Symptom changes were quantified using the Liebowitz Social Anxiety Scale (LSAS), Social Phobia Inventory (SPIN), Patient Health Questionnaire-9 (PHQ-9), Generalized Anxiety Disorder 7-Item Scale (GAD-7), and Quality of Life Enjoyment and Satisfaction Questionnaire Short Form (Q-LES-Q-SF). Clinical improvements were accompanied by increased alpha band power, decreased delta band power, and an increased alpha-delta ratio on serial spectral resting-state EEGs. Our findings suggest that PrTMS may warrant further investigation as a potential adjunctive treatment for SAD. Further large-scale, blinded, and randomized studies are warranted to validate our observations on the feasibility of PrTMS for SAD.

## 1 Introduction

The study focuses on personalized repetitive transcranial magnetic stimulation (PrTMS) as a modality to improve symptoms in a patient with social anxiety disorder (SAD), performance-only subtype. SAD is one of the most common mental disorders, wherein few seek treatment due to fear of engaging in social situations. Among those treated, first-line treatments, including behavioral and pharmacotherapy, yield response in 34% to 65% of patients, with continued remission reported in up to 35% (1). This highlights a potential role for adjunctive modalities to enhance treatment response rates in patients with SAD.

Repetitive transcranial magnetic stimulation (rTMS) is a non-invasive intervention that modulates cortical excitability and promotes adaptive neuroplasticity, with growing evidence supporting its utility in anxiety-related disorders. rTMS targeting the prefrontal cortex has been shown to reduce anxiety severity, improve emotional regulation, and modulate threat-processing networks implicated in SAD and related anxiety disorders (2–4). However, treatment response remains variable, and optimal stimulation parameters for SAD have not yet been firmly established.

PrTMS is a novel form of neuromodulation that delivers low-intensity stimulation across multiple scalp sites, rather than high-intensity stimulation of a single site, e.g. left dorsolateral prefrontal cortex (DLPFC), seen in many conventional rTMS protocols. The primary innovation of PrTMS compared to standard rTMS lies in its adaptive protocol generation, in which stimulation parameters, including amplitude, frequency, train duration, and intertrain interval, are iteratively updated based on data from weekly spectral electroencephalograms (EEGs) and psychometric questionnaires, using a proprietary software (PeakInput™) (5). It has shown promising results in patients with major depressive disorder (MDD), generalized anxiety disorder (GAD), post-traumatic stress disorder (PTSD), insomnia, concussion, and autism spectrum disorder (ASD) (6). Despite widespread use of validated anxiety questionnaires in SAD, these tools are rarely incorporated into neuromodulation therapies. Conventional rTMS protocols typically apply fixed stimulation parameters without adjusting for longitudinal symptom and EEG variability (28), which may limit treatment optimization. This represents a gap in neuromodulation that this study aims to explore.

## 2 Case presentation

Patient is an adult male with a longstanding history of performance-related anxiety starting in early adolescence. He reported heightened fear, hyperarousal, enhanced physiologic tremors, and “freezing” during evaluative situations such as test-taking, oral recitations, musical performances, and public speaking. His condition had resulted in marked anticipatory distress and impaired quality of life. In addition, he reported persistent concern and worry of acting in ways which may be negatively judged and potentially lead to embarrassment. Liebowitz Social Anxiety Scale (LSAS) was administered, and showed a total score of 57 (Fear: 32; Avoidance: 25), indicating moderate social anxiety. The patient denied significant anxiety in social situations outside of performance-based or evaluative settings, consistent with a diagnosis of social anxiety disorder, performance-only subtype. PrTMS was therefore recommended in early 2025 to alleviate his symptoms, decrease functional impairment, and improve overall quality of life.

## 3 Methods

### 3.1 Subject

The subject was selected based on a clinical diagnosis of SAD, established according to Diagnostic and Statistical Manual of Mental Disorders, 5th Edition (DSM-5) criteria (10). Baseline psychiatric evaluation included a structured clinical interview conducted by a board-certified psychiatrist, along with standardized questionnaires (PHQ-9 and GAD-7), to systematically assess for psychiatric comorbidities. There was no clinical evidence of MDD or GAD at baseline. The subject had not previously taken any psychotropic medications nor participated in cognitive behavioral therapy (CBT) for his symptoms. After discussion of these first-line options, the patient expressed a preference for neuromodulation-based modalities and elected to pursue PrTMS. He had no contraindications to TMS, such as ferromagnetic implants within 12 inches of the head, cardiac pacemaker, implantable cardioverter defibrillator, and history of seizures or brain lesions (7). The subject was recommended once-daily PrTMS sessions for a duration of 6 weeks, with the possibility of a 6-week extension to consolidate gains. The patient completed 50 sessions over a 12-week treatment course.

### 3.2 Personalized repetitive transcranial magnetic stimulation treatment

PrTMS was administered by certified neurotechnologists using a proprietary frequency algorithm (PeakLogic, Inc., San Diego, CA) installed in the Apollo TMS Therapy System. Stimulation targets included the following locations on the scalp: Cz (central midline), Fz (frontal midline), F3 (left dorsolateral prefrontal cortex), F4 (right dorsolateral prefrontal cortex), and Fpz (prefrontal midline). The total treatment time was approximately 30 minutes per session. Stimulation intensity ranged from 25% to 60% of the resting motor threshold (RMT), with a stimulus frequency range of 8–12 Hz. Pulses were delivered in 10–15 second trains, with an intertrain interval of 10–30 seconds. Stimulation parameters, including amplitude, frequency, train duration, and intertrain interval, were adjusted on a weekly basis via computerized algorithm (PeakInput™), which generates treatment protocols based on weekly spectral EEG measurements and symptom-based questionnaires (5). This allowed for region-specific modulation across stimulation sites. However, exact session-by-session parameter changes were not readily available for *post hoc* quantification. Therefore, analysis focused on longitudinal EEG and clinical outcomes rather than parameter changes. All sessions were completed without any adverse events.

### 3.3 Spectral EEG measurement and analysis

**Figure 1:**
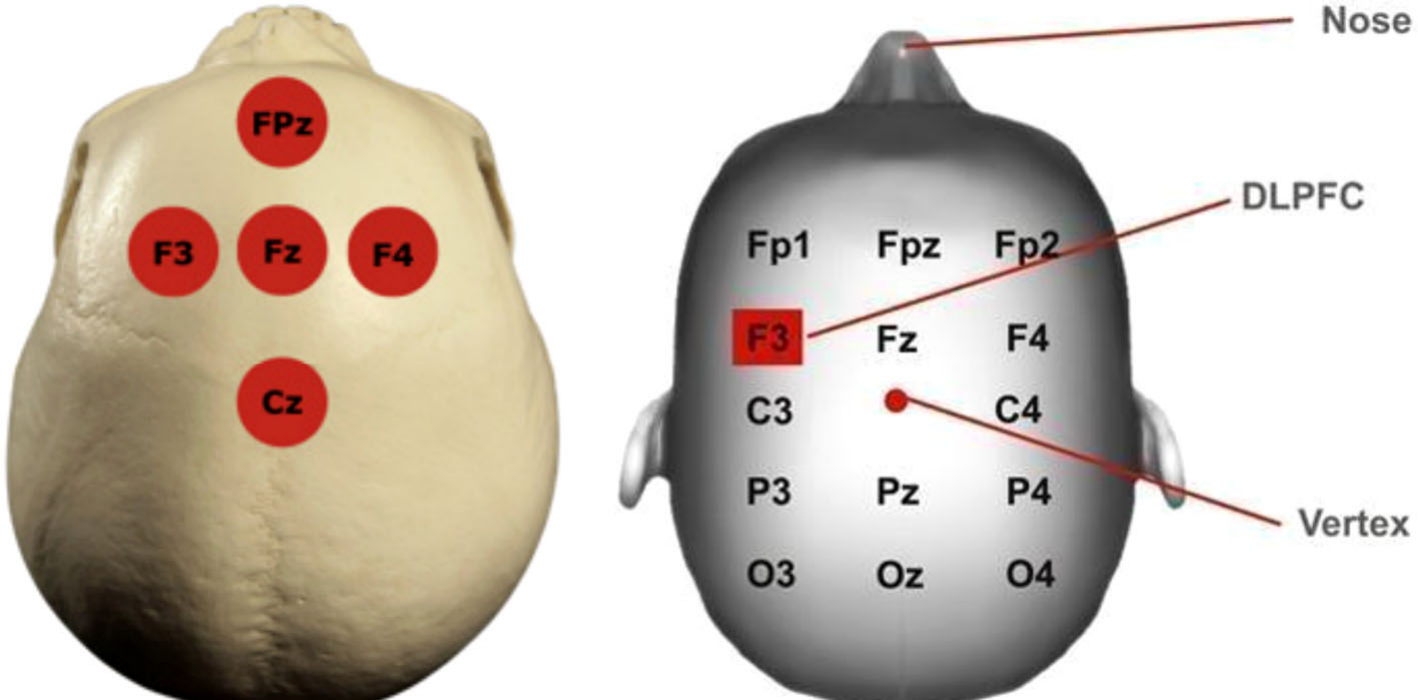
PrTMS locations according to the 10–20 system.

Resting-state EEG was performed weekly and at 3-month naturalistic follow-up. During 5-minute recordings, the subject was seated comfortably in a dimly lit room, and instructed to close his eyes and maintain a calm aware state. Readings were obtained using a CGX high-impedance dry electrode headset, with electrodes positioned according to the 10–20 system (**Figure 1**).

File outputs (.eeg,.vhdr,.vmrk) of pre-treatment, midpoint, final treatment, and follow-up EEGs were imported into an open source software (MNE-Python v1.11) for offline analysis (25). Frontal leads (Fp1, Fp2, Fz, F3, F4, F7, F8) were selected on the basis of prior studies linking frontal alpha-delta balance to altered top-down regulatory processes in SAD (14, 15). To attenuate noise, signals were notch filtered at 60 Hz, and a bandpass filter of 1–40 Hz was applied using a Butterworth IIR filter (26). Artifact removal was done for channels with excessive peak-to-peak variability in amplitude. Transient high-amplitude segments > 300 µV, and transient flat segments < 1 µV, were excluded from analysis. Power spectral density (PSD) was estimated using Welch’s method, with 4-second windows, 50% overlap, and Fast Fourier Transform (FFT) length equal to the window length. Absolute band power (µV²) was calculated by integrating the PSD within the alpha (8–13 Hz) and delta (1–4 Hz) bands. Median values for alpha power, delta power, and alpha-delta ratio across frontal leads were calculated. Raw EEG data were used in real-time by PeakInput to modify the amplitude, frequency, train duration, and intertrain interval of each treatment location on a weekly basis, while frontal alpha and delta metrics were calculated *post hoc*. Absolute power values are system- and preprocessing-dependent, and can vary across studies due to differences in band power analysis and physical features (e.g. hair volume affecting contact quality and impedance). As a result, we interpret the data in terms of longitudinal change, rather than comparing normative values across different studies (25–27). To this end, relative band power (RBP) in the alpha and delta ranges were calculated (RBP = P_8-13_/P_1–40_ and P_1-4_/P_1-40_). RBP represents the proportion of total EEG power across the 1–40 Hz range (P_1-40_), and is bounded between 0 and 1.

### 3.4 Psychometric questionnaire administration

Symptoms were monitored using standardized psychometric questionnaires. The Liebowitz Social Anxiety Scale (LSAS) and Social Phobia Inventory (SPIN) were administered as reliable measures of social anxiety (21, 22). Additionally, the Quality of Life Enjoyment and Satisfaction Questionnaire Short Form (Q-LES-Q-SF) was administered as a validated measure of health-related quality of life and overall satisfaction (23). These surveys were given on the initial day, at midpoint, and on the day of the final session to monitor longitudinal symptom changes. Lastly, the Patient Health Questionnaire-9 (PHQ-9) and Generalized Anxiety Disorder-7 (GAD-7) were administered weekly as reliable psychometric tools to monitor for emergent or comorbid depression and generalized anxiety symptoms (24). All surveys were re-administered after a 3-month naturalistic follow-up period to evaluate durability of treatment response. All tests were administered and scored according to published guidelines.

## 4 Discussion

SAD is one of the most common mental health disorders, with a 12-month prevalence of up to 8%, and a lifetime prevalence of up to 13% (1). It is characterized in the DSM-5 by marked fear or anxiety of 1 or more social situations in which an individual is exposed to potential scrutiny. These situations include social interactions, being observed, and performing in front of others. Individuals with SAD also fear that they will act in ways that may be negatively evaluated, leading to humiliation, embarrassment, or rejection. Triggers almost always provoke heightened anxiety that is out of proportion to the actual threat posed. The DSM-5 also includes a performance-only specifier, which is considered when fear is limited to evaluative situations only, distinguishing it from broader presentations. Median onset is between ages 13 and 15, and unlike transient situational anxiety, SAD involves persistent dysfunction in social, occupational, and academic domains. These features must persist for at least 6 months for clinical diagnosis (8–10). Individuals may also exhibit autonomic symptoms such as tremors, palpitations, flushing, and diaphoresis, which contribute to impaired daily function and quality of life (11). These findings are associated with maladaptive neuroplasticity, which further promotes fear learning (12).

Neurobiologically, SAD is linked to dysregulation of circuits involving the prefrontal cortex, amygdala, insula, hippocampus, and anterior cingulate cortex, contributing to exaggerated threat perception and impaired top-down emotional regulation (13). EEG studies have examined alterations in alpha band activity in individuals with SAD. In a longitudinal study of 103 adolescents, those classified as having stable high social anxiety were more likely to exhibit lower prefrontal alpha-delta ratios compared to those with stable low social anxiety. This decreased ratio may serve as a potential biomarker for stable, long-term shyness and avoidance behaviors in younger populations (14, 15). In addition, while frontal alpha asymmetry between the left and right hemispheres has been associated with dysregulation of prefrontal cortical activity in GAD (16), findings are mixed in SAD (17, 18).

**Figure 2:**
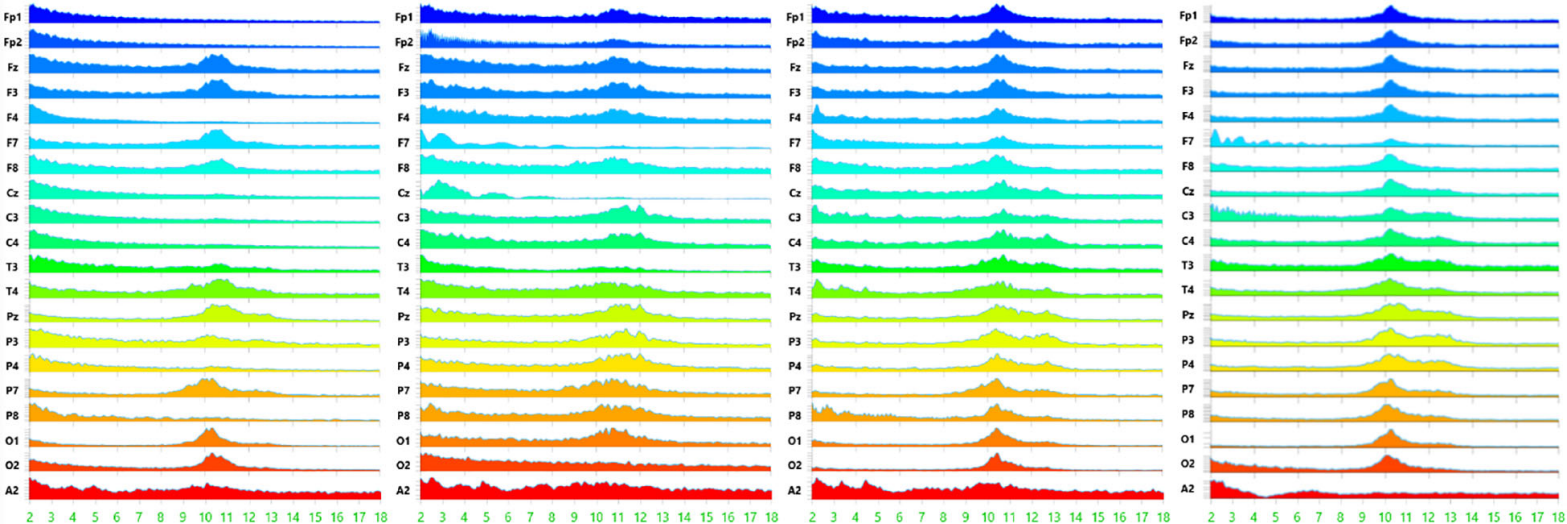
Spectral EEGs pre-treatment (1/7/25), at midpoint (2/18/25), on final session (4/1/25), and at 3-month follow-up (7/1/25).

**Figure 3:**
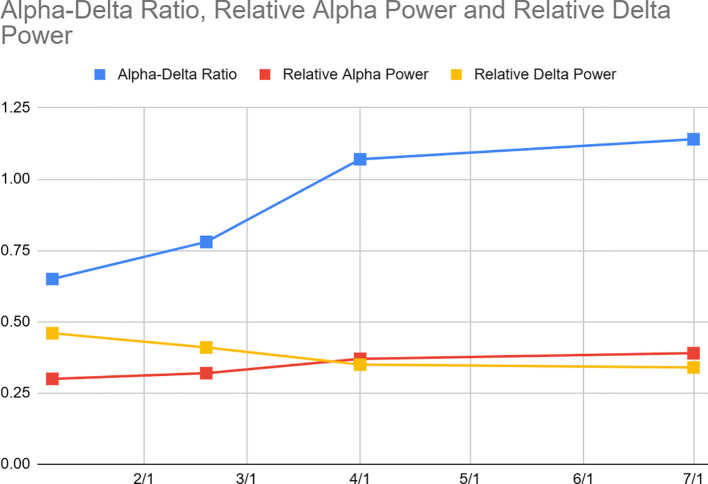
Increasing relative alpha power, decreasing relative delta power, and increasing alpha-delta ratio on serial resting-state EEGs.

**Figure 4:**
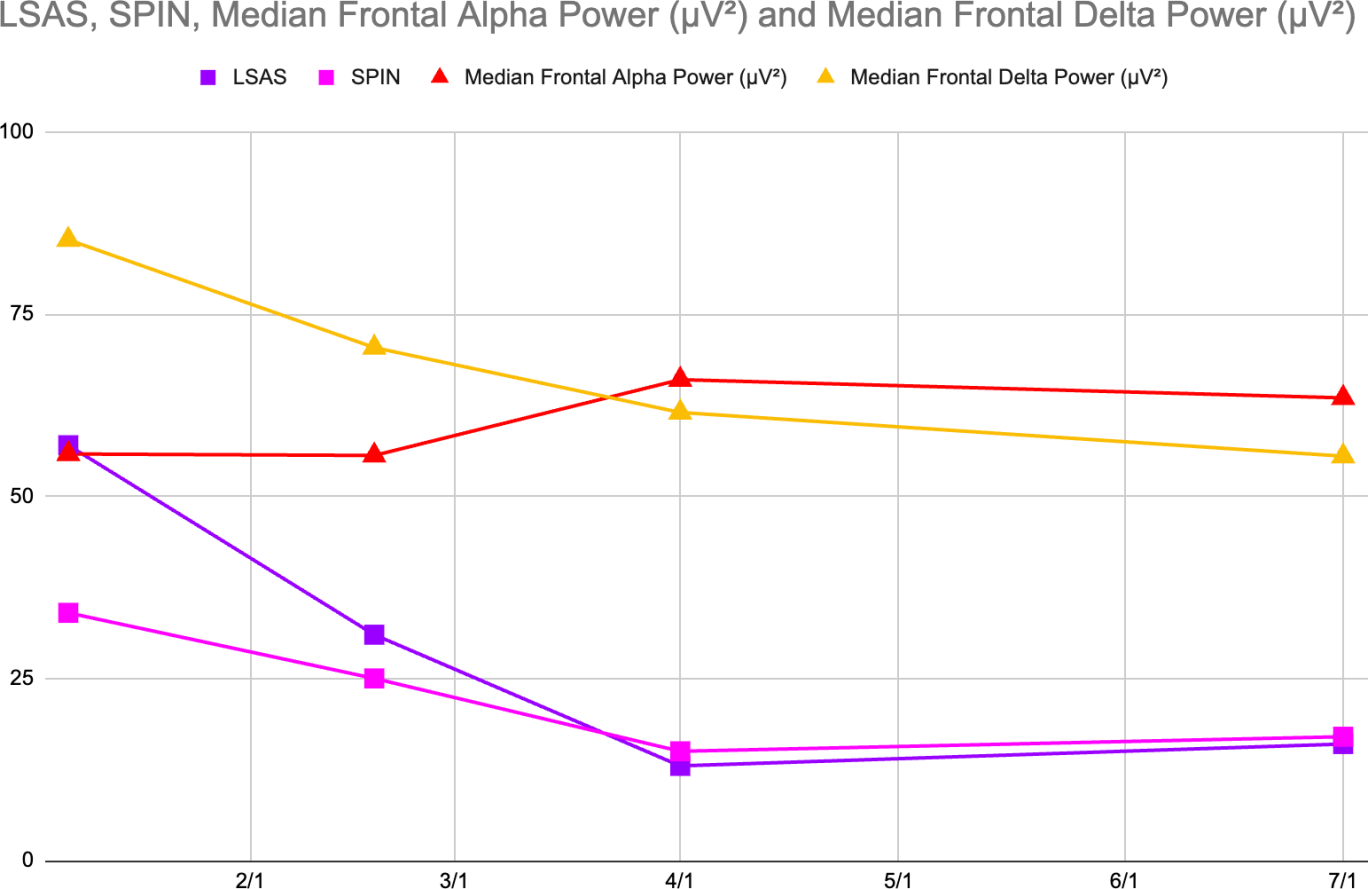
Decreasing LSAS and SPIN scores coinciding with increasing median frontal alpha power and decreasing median frontal delta power on serial resting-state EEGs.

This case highlights the potential utility of longitudinal EEGs and psychometric assessments to not only monitor treatment response, but also to inform treatment protocols, in patients with social anxiety and other chronic mental health conditions. Alpha oscillations, particularly in frontal leads (Fz, F3, F4, F7, F8, Fp1, and Fp2), are commonly interpreted as indices of cortical efficiency, calm alertness, creativity, focus, and top-down control (19, 20). Over the treatment course and follow-up period, serial spectral resting-state EEG readings demonstrated visually increased alpha band and decreased delta band amplitude across multiple leads, accompanied by improved alpha symmetry (e.g. Fp1 vs. Fp2, F3 vs. F4). Baseline to follow-up EEG demonstrated sharper alpha peaks centered at approximately 10 Hz (**Figure 2**). There was a gradual increase in median and relative frontal alpha power, and a gradual decrease in median and relative frontal delta power, with a resulting increase in alpha-delta ratio (**Figures 3**, **4**, **Table 1**). These findings reflect a shift from delta to alpha predominance in frontal regions on resting-state EEGs, which coincided with psychometric improvements. LSAS scores decreased by 71.9%, and SPIN scores by 50% (**Figures 4**, **Tables 2**, **3**], with the participant reporting improved composure and decreased autonomic symptom severity during evaluative situations. Q-LES-Q-SF scores increased by 28.8% from baseline (**Table 4**), coinciding with subjective improvements in occupational functioning, social relationships, household activities, overall sense of well-being, and future outlook. Lastly, PHQ-9 and GAD-7 scores remained stable and non-indicative of a potential MDD or GAD diagnosis throughout the 6-month study period (**Table 5**). Overall, this report is strengthened by its longitudinal within-subject design, along with integration of objective EEG metrics and validated psychometrics into an adaptive neuromodulation framework.

**Table 1:** Frontal alpha and delta metrics.

EEG Metrics	1/7/2025 (Initial)	2/18/2025 (Midpoint)	4/1/2025 (Final)	7/1/2025 (3-Mo. FU)
Median Frontal Alpha Power (µV²)	55.8	55.6	66.0	63.5
Median Frontal Delta Power (µV²)	85.2	70.4	61.5	55.5
Alpha-Delta Ratio	0.65	0.78	1.07	1.14
P_1-40_ (µV²)	185.3	171.1	177.8	162.4
Relative Alpha Power	0.30	0.32	0.37	0.39
Relative Delta Power	0.46	0.41	0.35	0.34

**Table 2:** LSAS scores.

LSAS	1/7/2025 (Initial)	2/18/2025 (Midpoint)	4/1/2025 (Final)	7/1/2025 (3-Month FU)
Raw score	57	31	13	16
Breakdown	32 (fear), 25 (avoidance)	12 (fear), 19 (avoidance)	5 (fear), 8 (avoidance)	7 (fear), 9 (avoidance)
Interpretation	Moderate SAD	Mild SAD	Not indicative of SAD	Not indicative of SAD

**Table 3:** SPIN scores.

SPIN	1/7/2025 (Initial)	2/18/2025 (Midpoint)	4/1/2025 (Final)	7/1/2025 (3-Month FU)
Raw score	34	25	15	17
Interpretation	Moderate SAD	Mild SAD	Not indicative of SAD	Not indicative of SAD

**Table 4:** Q-LES-Q-SF scores.

Q-LES-Q-SF	1/7/2025 (Initial)	2/18/2025 (Midpoint)	4/1/2025 (Final)	7/1/2025 (3-Month FU)
Raw score	43	50	58	52
% Maximum	52%	64%	78%	67%

**Table 5:** Weekly PHQ-9 and GAD-7 scores.

PHQ-9 and GAD-7 Scores	1/7	1/14	1/21	1/28	2/4	2/11	2/18	2/25	3/4	3/11	3/18	3/25	4/1	7/1
PHQ-9	4	3	4	2	1	0	1	2	2	2	1	0	2	3
GAD-7	7	5	6	3	4	5	3	4	2	3	2	1	2	3

Between the final treatment session and the 3-month naturalistic follow-up, there was mild attenuation of EEG and psychometric score improvements, although values remained markedly improved compared to baseline. Clinically, the patient reported durability of symptom improvements and preserved functional ability throughout this period. These findings may potentially be attributed to stabilization of neuromodulation effects, along with partial regression due to the absence of continued neurostimulation. These underscore the need for further studies to better characterize the durability of clinical and EEG changes over time.

## 5 Limitations

The use of adaptive neuromodulation for social anxiety was limited to a single case study. Its feasibility must be further studied using large-scale, blinded, and randomized trials in order to validate findings. Additionally, the use of self-reported psychometric questionnaires is subject to response bias and placebo effects. To contextualize these subjective outcomes, serial longitudinal EEGs were administered as a more objective measure of neurophysiologic activity. These preliminary results are best characterized as hypothesis-generating groundwork for future studies.

## 6 Patient perspective

I’ve been dealing with situational anxiety since my early teens. Situations where I had to perform or be evaluated, like presentations and exams, would cause immense anxiety, along with physical symptoms like palpitations, sweating, and trembling. I would worry too much about making mistakes or being judged negatively, and even when I would do better than I expect, there would still be some worry and self-blame over what I could have done better.

After starting my treatments, I began to notice gradual changes. Situations that used to trigger overwhelming anxiety began to feel less intense and more manageable. I felt less overwhelmed, and noticed myself being more able to communicate, articulate, and be evaluated by others without freezing as much. I also found myself being more willing to participate in situations or events that I would have previously avoided. While I still have some anxiety in high-pressure situations, it feels much more manageable than before, and I have found myself having less physical symptoms and being more willing to engage while being put on the spot.

## 7 Conclusion

Adaptive neuromodulation strategies such as PrTMS are understudied for the treatment of SAD, which is characterized by dysregulated neural activity in the frontal and limbic cortices. This dysregulation may not be optimally targeted by fixed, single-site stimulation protocols such as standard left DLPFC stimulation. In this context, EEG- and psychometric-guided treatment protocols provide a structured and potentially objective framework for monitoring symptom changes over time. While causation cannot be drawn from a single case, the coincidence of psychometric and spectral findings warrants further large-scale, blinded, and randomized studies to better understand the role of EEG-guided, psychometric-informed protocols for individuals with SAD.

## Data Availability

The original contributions presented in the study are included in the article/supplementary material. Further inquiries can be directed to the corresponding author.
